# Characterization and localization of cyclin B3 transcript in both oocyte and spermatocyte of the rainbow trout (*Oncorhynchus mykiss*)

**DOI:** 10.7717/peerj.7396

**Published:** 2019-07-24

**Authors:** Wenzhi Guan, Liangjie Qiu, Bo Zhang, Jianbo Yao, Qing Xiao, Gaofeng Qiu

**Affiliations:** 1 Key Laboratory of Freshwater Aquatic Genetic Resources, Ministry of Agriculture; National Demonstration Center for Experimental Fisheries Science Education; Shanghai Engineering Research Center of Aquaculture, Shanghai Ocean University, Shanghai, China; 2 College of Life Science and Technology, Huazhong Agricultural University, Wuhan, China; 3 Division of Animal and Nutritional Sciences, West Virginia University, Morgantown, USA; 4 College of Fisheries, Huazhong Agricultural University, Wuhan, China

**Keywords:** Cyclin B3, Oogenesis, Rainbow trout, Expression, Spermatogenesis

## Abstract

B-type cyclins are regulatory subunits with distinct roles in the cell cycle. To date, at least three subtypes of B-type cyclins (B1, B2, and B3) have been identified in vertebrates. Previously, we reported the characterization and expression profiles of cyclin B1 and B2 during gametogenesis in the rainbow trout (*Oncorhynchus mykiss*). In this paper, we isolated another subtype of cyclin B, cyclin B3 (CB3), from a cDNA library of the rainbow trout oocyte. The full-length CB3 cDNA (2,093 bp) has an open reading frame (1,248 bp) that encodes a protein of 416 amino acid residues. The CB3 transcript was widely distributed in all the examined tissues, namely, eye, gill, spleen, brain, heart, kidney, stomach, skin, muscle, and, especially, gonad. Northern blot analysis indicated only one form of the CB3 transcript in the testis and ovary. *In situ* hybridization revealed that, in contrast to cyclin B1 and B2 transcripts, CB3 transcripts were localized in the oocytes, spermatocytes, and spermatogonia. These findings strongly suggest that CB3 plays a role not only as a mitotic cyclin in spermatogonial proliferation during early spermatogenesis but also during meiotic maturation of the spermatocyte and oocyte in the rainbow trout.

## Introduction

The rainbow trout (*Oncorhynchus mykiss*) is a freshwater salmonid species native to cold-water rivers and lakes of the Pacific Ocean in Asia and North America. Because of its high commercial value, the trout has been introduced to non-native waters worldwide, and it is the most important aquaculture salmonid species in many countries. In 1959, the trout was introduced to China from North Korea, and, to date, it is farmed in more than 20 provinces. Because the rainbow trout is easy to maintain, grows fast, and is tolerant to a wide range of environments, it is used as an experimental animal model for studies of reproduction and development. Usually, farmed rainbow trouts are incapable of breeding naturally in culture systems, and artificial propagation needs to be performed in trout hatcheries. The reproductive physiology and sex differentiation of the rainbow trout have been studied in detail (Bellaiche et al., 2014; Kiilerich et al., 2018; Nicol & Guiguen, 2011; Salem et al., 2013).

Gametogenesis is a developmental process regulated precisely by the mitotic and meiotic cell cycles. The eukaryotic cell cycle is driven by the temporally controlled activation of protein kinase complexes, which are composed of a cyclin as the regulating subunit and cyclin-dependent kinase (CDK) as the catalytically active component. Cyclins activate CDKs and thereby regulate the transition through the cell cycle (Sanchez & Dynlacht, 2005). On the basis of amino acid sequence similarities and differences in expression patterns during the cell cycle phases, cyclins are divided into multiple subfamilies. A- and B-type cyclins are distinguished by their distinct appearances in the S and G2 phases and their rapid proteolytic destruction during mitosis (Evans et al., 1983). The B-type cyclins are responsible for controlling the transition from G2 to mitosis. In vertebrates, at least three B-type cyclins, cyclin B1 (CB1), cyclin B2 (CB2) and cyclin B3 (CB3), have been found (Table 1). In fish, expressions of the three B-type cyclins have been well characterized. In the rainbow trout, CB1 and CB2 exhibit differential expression of the mRNA and protein during oogenesis and spermatogenesis (Qiu et al., 2008). CB1 has a major role in the regulation of meiotic maturation of oocytes and spermatocytes.

**Table 1:** The expression and potential roles of B-type cyclins in vertebrates.

B-type cyclins	Species	Expression	Potential roles	Reference
CB1	*Oncorhynchus mykiss*	In previtellogenic and mature oocytes; in spermatogonia and spermatocytes	In the regulation of meiotic maturation of oocyte and spermotocyte	Qiu et al. (2008)
CB1	*Oryzias latipes*	In spermatogonia and spermatocytes at prophase and metaphase	Controlling the meiotic cell cycle	Mita et al. (2000)
CB1	Human	In the perinuclear cytoplasm and nucleus	Associates with the chromosomes and the mitotic spindle	Pines & Hunter (1991)
CB2	Mouse	In the oocytes	Compensation for Cyclin B1 in oocyte meiosis I	Li et al. (2018)
CB2	*Xenopus sp.*	In/around the germinal vesicle of oocyte	Essential for bipolar spindle formation in the oocytes	Yoshitome et al. (2012)
CB2	*Oncorhynchus mykiss*	In immature oocytes, spermatogenesis	In the differentiation from spermatid to spermatozoa	Qiu et al. (2008)
CB2	*Oryzias latipes*	All stages of spermatogenic cells	Involved in process(es) other than meiosis	Mita et al. (2000)
CB3	*Danio rerio*	In the type A and early type B spermatogonia	Used as markers of spermatocytes and early spermatogonia	Ozaki et al. (2011)
CB3	*Anguilla japonica*	In spermatogonia but no in spermatocyte and sperm	In spermatogonial proliferation (mitosis), but not in meiosis	Kajiura-Kobayashi, Kobayashi & Nagahama (2004)
CB3	Mouse	From the onset of the first meiotic prophase to the end of pachytene stage	In early meiotic prophase I	Nguyen et al. (2002)
CB3	Human	Not only in S and G2/M cells but also in G0 and G1	A dominant-negative function in competition with activating cyclins in G0 and the G1 phase of the cell cycle	Tschoep et al. (2006)

Since cyclin B3 (CB3) was initially cloned from a chicken cDNA library, it has been identified in a wide variety of model species, such as worm, fruit fly, mouse, and human (Gallant & Nigg, 1994; Jacobs, Knoblich & Lehner, 1998; Kajiura-Kobayashi, Kobayashi & Nagahama, 2004; Kreutzer et al., 1995; Nguyen et al., 2002). In the fruit fly (*Drosophila melanogaster*), CB3 is expressed in both mitotic and meiotic cells. Previous gene inactivation studies have shown that CB3 is unessential for mitosis but required for fertility. CB3-deleted female individuals were sterile because the oocytes could not complete meiosis I (Chen et al., 2018; Jacobs, Knoblich & Lehner, 1998). In the nematode *Caenorhabditis elegans*, CB3 may be required for oocyte meiosis or early development, as it is strongly expressed in the female germline (Kreutzer et al., 1995). Similarly, in mammals, CB3 mRNA and protein were detected in prepachytene spermatocytes, especially at leptotene and zygotene stages, and nests of female oocytes during embryonic development (Nguyen et al., 2002). In the Japanese eel (*Anguilla japonica*), isolation of CB3 cDNA and its increased expression were reported during hormonally induced spermatogenesis; the data suggest that Japanese eel CB3 is specifically involved in spermatogonial proliferation (mitosis), but not in meiosis (Kajiura-Kobayashi, Kobayashi & Nagahama, 2004). This result is not consistent with the findings of previous studies in mammals. To understand the role of CB3 in the maturation of germ cells in fish, we cloned rainbow trout CB3 and examined its expression profiles during oogenesis and spermatogenesis.

## Materials and Methods

### Animals and tissue collection

Rainbow trouts were collected every month from a local hatchery during the breeding season. All handling of fishes were conducted in accordance with guidelines on the care and use of animals for scientific purposes set up by the Institutional Animal Care and Use Committee (IACUC) of Shanghai Ocean University (SHOU-DW-2017021), Shanghai, China. Gonad and somatic tissues were rapidly dissected and frozen in liquid nitrogen and stored at −80 °C until use. A small part of the gonad tissue was also fixed in Davidson’s fixative and 4% paraformaldehyde in phosphate-buffered saline for tissue sectioning. Embryos were generated by artificial insemination and incubated in recirculating water at a low temperature (approximately 13 °C). Developing embryos at various stages were sampled as described previously (Ramachandra et al., 2007).

### Total RNA extraction

Total RNA was isolated from gonad and somatic tissues by using TRIzol reagent (Invitrogen, Carlsbad, CA), according to the manufacturer’s protocol. Then, the RNA was treated with DNase I (Ambion Inc., Austin, TX, USA) to remove contaminant genomic DNA. Finally, the quality of each RNA sample was checked with 1% agarose gel electrophoresis.

### cDNA clone isolation for CB3

A cDNA clone for rainbow trout CB3 was isolated from the trout oocyte cDNA library (Yao, Higgins & Rexroad, 2005) on the basis of EST information. The clone was fully sequenced in both directions.

### Regular RT-PCR and quantitative real-time RT-PCR

About 500 ng of total RNA was reverse-transcribed to single-strand cDNA by using the Prime Script RT reagent kit (TaKaRa, Kusatsu, Japan). Regular PCR was performed to examine tissue distribution of CB3 transcripts using a pair of CB3 primers and β-actin was employed as an internal control (Table 2). The PCR protocol was as follows: 35 cycles of denaturation at 94 °C for 30 s, annealing at 55 °C for 30 s, and extension at 72 °C for 30 s. For real-time quantitative PCR (qPCR), another pair of CB3 primers was designed within the coding regions (Table 2). Histone H2a was selected as the internal reference because of its relatively stable expression during early embryo development (Robert et al., 2002). qPCR was performed with SYBR Green Premix ExTaq (TaKaRa, Kusatsu, Japan). Reaction mixtures (25 µl) were pre-incubated for 15 min at 95 °C to activate HotStartTaq DNA polymerase, and the PCR protocol was as follows: 40 cycles of denaturation at 94 °C for 30 s, annealing at 56 °C for 30 s, and extension at 72 °C for 30 s. Standard curves were created using 10-fold serial dilutions of the corresponding plasmid with inserts for the target and reference genes. The triplicate fluorescence intensities of each sample were measured using crossing-point (Ct) values and converted to fold differences with the relative CT (2^−ΔΔCT^) method.

**Table 2:** Primer sequences used in this study.

Primer name	Primer sequence (5′-3′)	Usage
CB3-qPCR-F	AGGTTCACATCAGCCTTCCA	Real-time PCR
CB3-qPCR-R	GCTTCTCCTCCTCCTGACTC	
CB3-F	GGACAGCCTTCGTAGACCTCAC	Regular RT-PCR
CB3-R	AGCTCAAAGTTCTCCTGCACCT	
Histone H2a-F	TCCCCAAGAAGACTGAGAAGG	Real-time PCR
Histone H2a-R	TTTGTTGAGCTAGGTGGTTGG	
*β*-Actin-F	AAGTGTGACGTGGACATCCGT	Regular RT-PCR
*β*-Actin-R	TAATCCGCTGCTTCACCGTTC	

### Preparation of digoxigenin-labeled RNA probes

A cDNA fragment of rainbow trout CB3 was amplified with gene-specific primers (Table 2) and cloned using the TOPO TA cloning kit (Invitrogen, Carlsbad, CA, USA). The plasmid clones were used as templates to synthesize digoxigenin (DIG)-labeled RNA probes with the DIG RNA labeling kit (Roche Diagnostics, Indianapolis, IN, USA).

### Northern blot analysis

Northern blot analysis was conducted as described previously (Qiu et al., 2008). Briefly, about 3 µg each of mRNA from the ovary and testis was separated by electrophoresis on a denaturing 1.2% agarose gel containing 6% formaldehyde. After electrophoresis, the mRNA was transferred onto a nylon membrane (Amersham Pharmacia Biotech, Piscataway, NJ, USA) and hybridized with DIG-labeled RNA probes.

### *In situ* hybridization

The gonadal tissue sections were incubated with DIG-labeled RNA probes at 55 °C overnight. Then, the sections were washed with formamide (50%) in 2 × SSC and treated with RNase A (20 µg/ml) in NTE buffer, and the hybridization signals were detected using anti-DIG antibodies conjugated with alkaline phosphatase and the substrate NBT/BCIP (Roche Diagnostics, Indianapolis, IN, USA), as described previously (Qiu & Yamano, 2005).

## Results

### Cloning and sequencing of CB3 cDNA

**Figure 1:**
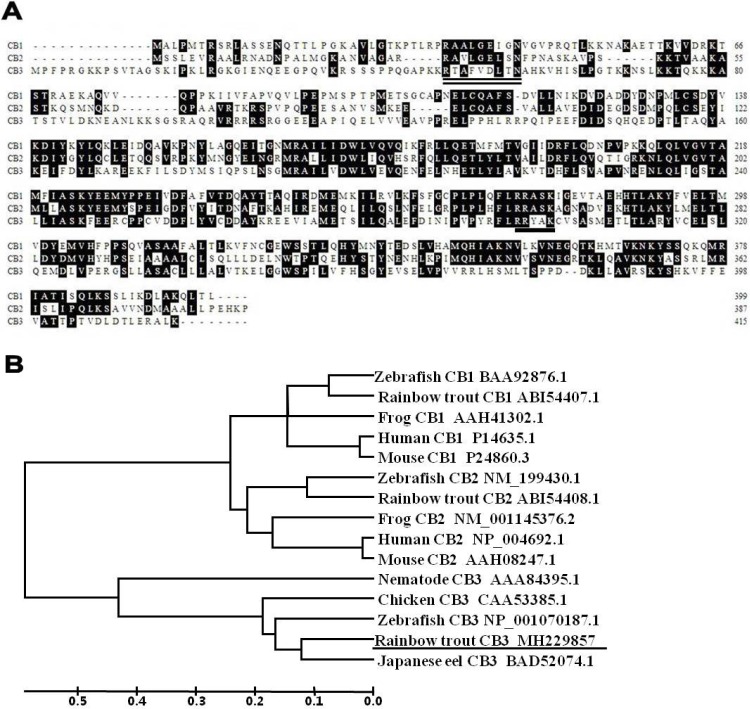
The deduced amino acid sequences and the phylogenetic tree. (A) Alignment of the deduced amino acid sequences for CB1, CB2, and CB3 in the rainbow trout. Conserved residues are shaded. The amino acid residues of putative pkA sites are underlined, and the putative destruction signal is denoted by a double underline. (B) Phylogenetic tree for CB1, CB2, and CB3 constructed with UPGMA (Kumar et al., 2001) by using the deduced amino acid sequences of CB1, CB2, and CB3 from GenBank. GenBank accession numbers for the sequences are listed on the right. The ruler indicates genetic distances.

A partial cDNA sequence for rainbow trout CB3 was identified from our collection of rainbow trout oocyte expressed sequence tags (ESTs). The corresponding cDNA clones were retrieved from the oocyte cDNA library plates. The clones were fully sequenced in both directions. After sequence analysis, the full-length cDNA sequence for rainbow trout CB3 was obtained. The cDNA is 2,131 bp in length and contains a polyadenylation signal (AATAAA) and poly (A) tail at the 3′ end (GenBank accession number: MH229857). The trout CB3 sequence contains a 1,246 bp open reading frame that potentially encodes a 416 amino acids protein (Fig. 1A) with a predicted molecular weight of 45 kDa. The original sequence proposed for the amino-terminal CB destruction box has a consensus of R-ALG(NDE)I-N, followed by a lysine-rich region (Glotzer, Murray & Kirschner, 1991; Hunt, 1991). The destruction box is present in the N-terminal region of the rainbow trout CB3 (double-underlined in Fig. 1A). The trout CB3 has four conserved amino acids, arginine, alanine, aspartic acid, and asparagine (R-A–D–N), like chicken CB3 (Gallant & Nigg, 1994), but it lacks other conserved amino acids. The predicted protein sequence also contains a putative pkA site (RRxxK; underlined in Fig. 1A), a characteristic of B-type cyclins (Minshull et al., 1989; Scheurlen, Hoffmeister & Schaller, 1996), strongly suggesting that the encoded protein exhibits cyclin activity.

Multiple alignments revealed that the trout CB3 was quite divergent when the predicted amino acids within the cyclin box were compared with previously reported trout CB1 and CB2 (Qiu et al., 2008). The entire protein displayed 48% identity with CB1 and 57% identity with CB2. However, the trout CB3 is more closely related to CB3 in other species. The protein sequence showed 68% similarity to zebrafish, 72% to chicken, 79% to nematode, and 86% to Japanese eel CB3. The phylogenetic tree revealed that the trout CB3 clustered with Japanese eel CB3 and then with zebrafish CB3 (Fig. 1B), suggesting high evolutionary conservation among the coding sequences in teleosts.

### Tissue distribution of CB3 transcripts

**Figure 2:**
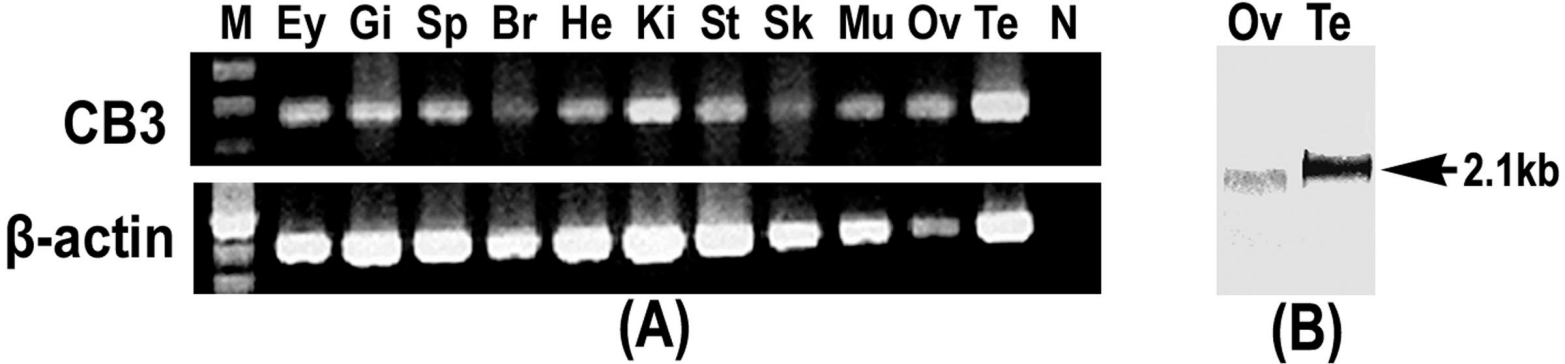
RT-PCR and Northern blot analysis of CB3. (A) RT-PCR analysis of CB3 mRNA in rainbow trout tissues. Gene-specific primer set used for the analysis is the same as that used for the amplification of cDNA fragments for RNA probe preparation (Table 2). Trout *β*-actin was used as the control for RNA quality. (B) Northern blot analysis of the CB3 transcript in mature ovary and testis of the rainbow trout. Each lane contains 3 µg of Poly(A)+ RNA. Three individuals were used in tissue distribution detection and each experiment was performed in triplicate. Ey, eye; Gi, gill; Sp, spleen; Br, brain; He, heart; Ki, kidney; St, stomach; Sk, skin; Mu, muscle; Ov, ovary; Te, testis; N, negative control; M, molecular weight standard.

Tissue expression of the trout CB3 transcript was examined using RT-PCR. The trout CB3 transcript was expressed in various tissues (Fig. 2A). It was clearly present in the testis and kidney, whereas it was detected at relatively low levels in the brain and skin. The northern blot analysis revealed that the CB3 transcript was detected as a single form of approximately 2.1 kb in both mature ovary and testis (Fig. 2B). The CB3 transcript size was consistent with the putative molecular weight of its corresponding cDNA sequence.

### Expression profile of CB3 mRNA during oogenesis and early embryogenesis

**Figure 3:**
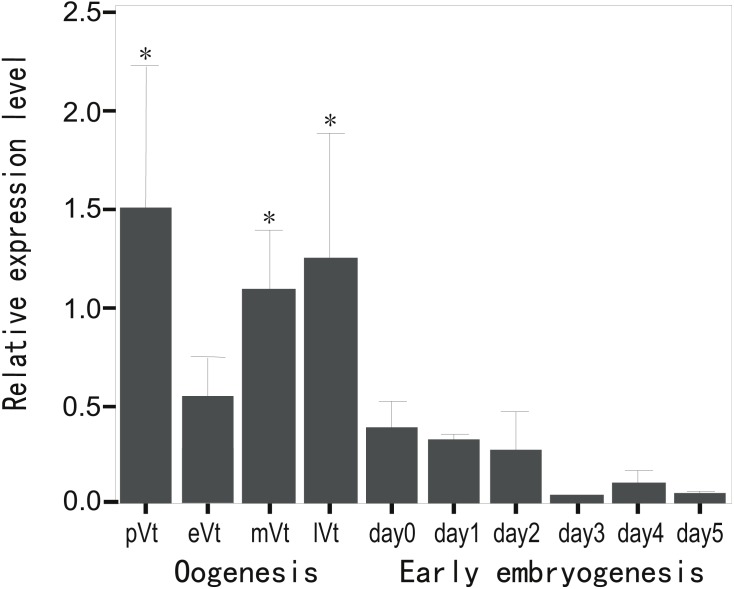
Quantification of CB3 transcripts during oogenesis and early embryogenesis by using real-time PCR. pVt, previtellogenesis; eVt, early vitellogenesis; mVt, middle vitellogenesis; lVt, late vitellogenesis; day 0, spawned eggs before fertilization; days 1–5, embryos 1–5 days post-fertilization. The quantities of target gene transcripts were normalized to that of histone H2a. Error bars represent SEM (*n* = 4).

To evaluate the expression profile of CB3 mRNA during oogenesis and early embryonic development, the relative level of CB3 mRNA in the ovaries at various stages and early embryos from day 1 to day 5 post-fertilization was quantified using histone H2a as the internal reference. The amount of CB3 mRNAs was significantly higher in the ovaries during previtellogenesis and middle and late vitellogenesis stages (*P* < 0.05) and lower during early vitellogenesis and embryogenesis (Fig. 3).

### Localization of the CB3 transcript in the ovary and testis

**Figure 4:**
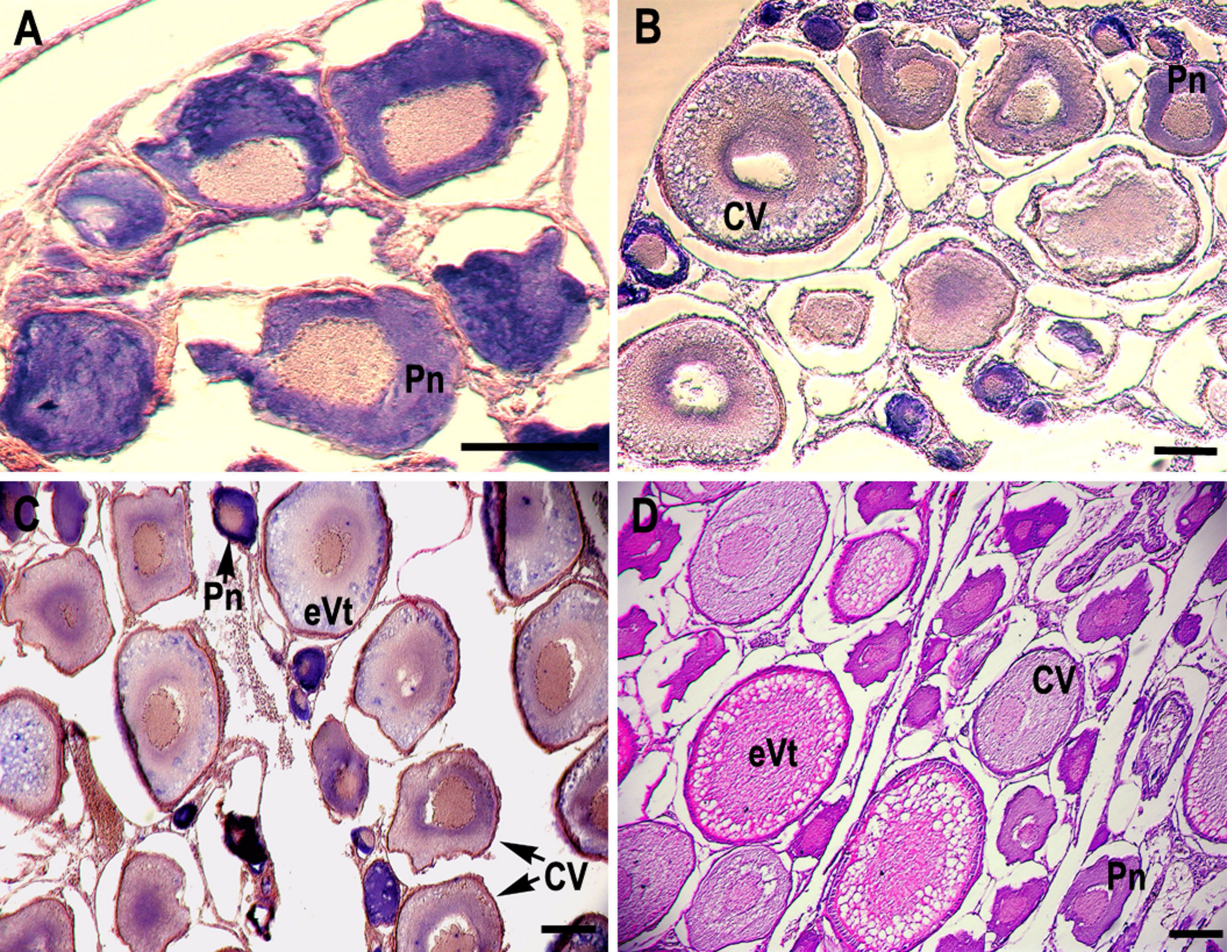
Localization of CB3 transcripts in rainbow trout ovary. (A–C) Transcripts were visualized by *in situ* hybridization with DIG-labeled anti-sense RNA probe. Positive signals are blue. Negative control using sense RNA probe was shown in supplement files. (D) Histological section stained with hematoxylin and eosin. Pn, perinucleolar oocyte (previtellogenic); CV, cortical vesicle (previtellogenic); eVt, early vitellogenic oocyte. Scale bar, 200 µm.

**Figure 5:**
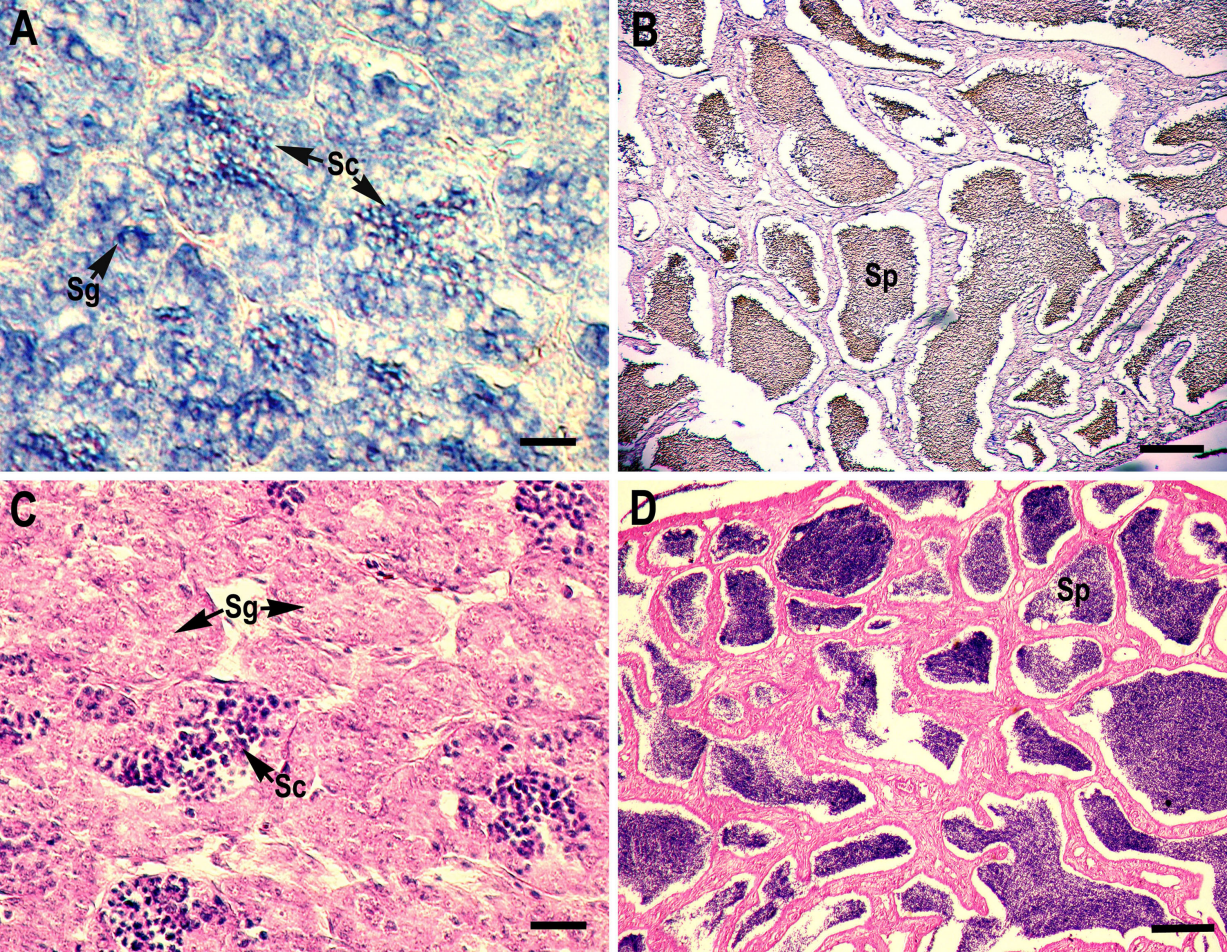
Localization of CB3 transcripts in the testes at early (A) and late spermatogenesis stages (B) of the rainbow trout. (A, B) Transcripts were visualized by *in situ* hybridization with DIG-labeled anti-sense RNA probe. Positive signals are blue. Negative control using sense RNA probe was shown in supplement files. (C, D) Histological section stained with hematoxylin and eosin. Sg, spermatogonia; Sc, spermatocytes; Sp, spermatids. Scale bar, 5 µm.

RNA *in situ* hybridization analysis was conducted using ovarian and testis tissue sections to determine the localization of the CB3 transcript in germ cells. In the ovarian tissue sections, strong hybridization signals (blue color) were detected in the ooplasm of perinucleolar oocytes, and weak signals were detected in the ooplasm of early vitellogenic oocytes (Fig. 4). In the testis, positive signals were detected in the spermatogonia and spermatocytes, but almost no signal was detected in the spermatids (Fig. 5).

## Discussion

Although CB3 has been extensively identified in a variety of species such as insects, chicken, and mammals (Table 1) (Gallant & Nigg, 1994; Jacobs, Knoblich & Lehner, 1998), there is limited information on CB3 in teleosts (Kajiura-Kobayashi, Kobayashi & Nagahama, 2004). In this study, we successfully isolated a full-length cDNA that encodes cyclin B3 from the rainbow trout. Despite high sequence homology with trout CB1 and CB2 (Fig. 1A), trout CB3 is more closely related to CB3 in other species (Fig. 1B), indicating the conservation of CB3 sequences. The northern blot analysis detected a single transcript for trout CB3 in the gonadal tissues, which is consistent with the data for the Japanese eel (Kajiura-Kobayashi, Kobayashi & Nagahama, 2004) and *Caenorhabditis elegans* (Kreutzer et al., 1995). However, at least three splice variants in the ORF and three variants in the 5′-UTR were found in human CB3 (Lozano et al., 2002). Human CB3 mRNA variant 1 has been detected in the skeletal muscle and variant 3, in the testis (Tschoep et al., 2006). Differences in the distribution of various forms of the transcripts are attributable to the existence of nuclear localization signals. Different forms of the CB3 transcript may reflect different regulatory modes during post-transcriptional processes.

The RT-PCR analysis revealed that the trout CB3 transcript was distributed homogeneously in various tissues. The CB3 mRNA level was remarkably high in the ovary and testis, indicating that CB3 may play an essential role in the gonads during gametogenesis. Quantitative real-time PCR analysis showed that the trout CB3 transcript level was higher during oogenesis than during early embryogenesis (Fig. 3). *In situ* hybridization further revealed that the CB3 transcript was mainly localized in the cytoplasm of previtellogenic oocytes (Figs. 4A–4C). These findings show that CB3 may be important for events that occur during early meiotic prophase I in the oocyte of the rainbow trout. In mammals, CB3 is a prepachytene meiotic cyclin, and it is important for events that occur during early meiotic prophase I.

The role of CB3 has been rarely characterized during spermatogenesis, when compared with oogenesis; however, the maturation promotion factor is assumed to have similar functions in this process. The expression of mouse CB3 mRNA was localized to the leptotene and zygotene spermatocytes. In mice, CB3 is expressed during the first spermatogenic wave, beginning at the onset of the first meiotic prophase and ending at the pachytene stage (Nguyen et al., 2002). Misexpression of CB3 leads to aberrant spermatogenesis, and downregulation of CB3 is required for normal spermatogenesis at the transition of zygotene to pachytene (Refik-Rogers, Manova & Koff, 2006). CB3 is also essential for promoting mitotic dynein functionality in the cell cycle (Deyter et al., 2010). In humans, CB3 mRNA is expressed in S and G2/M cells as well as in G0 and G1 (Tschoep et al., 2006). Enforced CB3 expression results in the accumulation of cells in the anaphase and, at even higher doses, G1 phase (Nguyen et al., 2002). Similarly, zebrafish CB3 was found in type A and early type B spermatogonia and germ cells in large cysts, possibly corresponding to spermatocytes at the preleptotene stage (Ozaki et al., 2011). However, Japanese eel CB3 expression is limited to spermatogonia, but absent in spermatocytes, during spermatogenesis, suggesting that Japanese eel CB3 is specifically involved in spermatogonial proliferation (mitosis), but not in meiosis (Kajiura-Kobayashi, Kobayashi & Nagahama, 2004). In the present study, *in situ* hybridization analysis revealed that rainbow trout CB3 transcripts are localized not only in the spermatogonia but also in spermatocytes (Fig. 5), indicating that CB3 may not only have a role as a mitotic cyclin during spermatogonial proliferation but also during the meiotic maturation of spermatocytes (like in mammals).

In summary, a full-length cDNA that encodes CB3 was isolated and characterized from the rainbow trout. The CB3 transcript in the rainbow trout, unlike in the Japanese eel, was localized in both spermatogonia and spermatocytes and mainly in previtellogenic oocytes, suggesting an important role for CB3 in the early events of mitosis and meiosis during spermatogenesis and oogenesis. This finding will be helpful for understanding the molecular mechanisms underlying gametogenesis in the rainbow trout and provide a valuable reference for better understanding of CB3 evolution.

##  Supplemental Information

10.7717/peerj.7396/supp-1 Supplemental Information 1 The full-length cDNA sequence for rainbow trout CB3 The CB3 cDNA is 2,131 bp in length and contains a 1,246 bp open reading frame that encodes 416 amino acids.

10.7717/peerj.7396/supp-2 Supplemental Information 2 RT-PCR analysis of CB3 mRNA in rainbow trout tissues (A) CB3 mRNA in rainbow trout tissues (B)Trout *β*-actin was used as the control for RNA quality. Ey, eye; Gi, gill; Sp, spleen; Br, brain; He, heart; Ki, kidney; St, stomach; Sk, skin; Mu, muscle; Ov, ovary; Te, testis; N, negative control; M, molecular weight standard.

10.7717/peerj.7396/supp-3 Supplemental Information 3 Northern blot analysis of the trout CB3 in the ovary (Ov) and testis (Te) The detection of an unique form of CB3 mRNA, of approximately 2.1 kb (arrow).

10.7717/peerj.7396/supp-4 Supplemental Information 4 A negative control of in situ hybridization analysis was performed using a sense probe

## Additional Information and Declarations

### DNA Deposition

The following information was supplied regarding the deposition of DNA sequences: Data is available at GenBank: MH229857.

### Data Availability

The following information was supplied regarding data availability: The full-length cDNA sequence for rainbow trout CB3 is available in the Supplemental Files.
