# Supplementary material for: Characterization and localization of cyclin B3 transcript in both oocyte and spermatocyte of the rainbow trout (Oncorhynchus mykiss)
Source: PeerJ. 2019 Jul 24;7:e7396. doi: 10.7717/peerj.7396 (PMC6660826; doi:10.7717/peerj.7396)
Supplement: Supplemental Information 2 — (A) CB3 mRNA in rainbow trout tissues (B)Trout β-actin was used as the control for RNA quality. Ey, eye; Gi, gill; Sp, spleen; Br, brain; He, heart; Ki, kidney; St, stomach; Sk, skin; Mu, muscle; Ov, ovary; Te, testis; N, negative control; M, molecular weight standard. [file peerj-07-7396-s002.pdf]

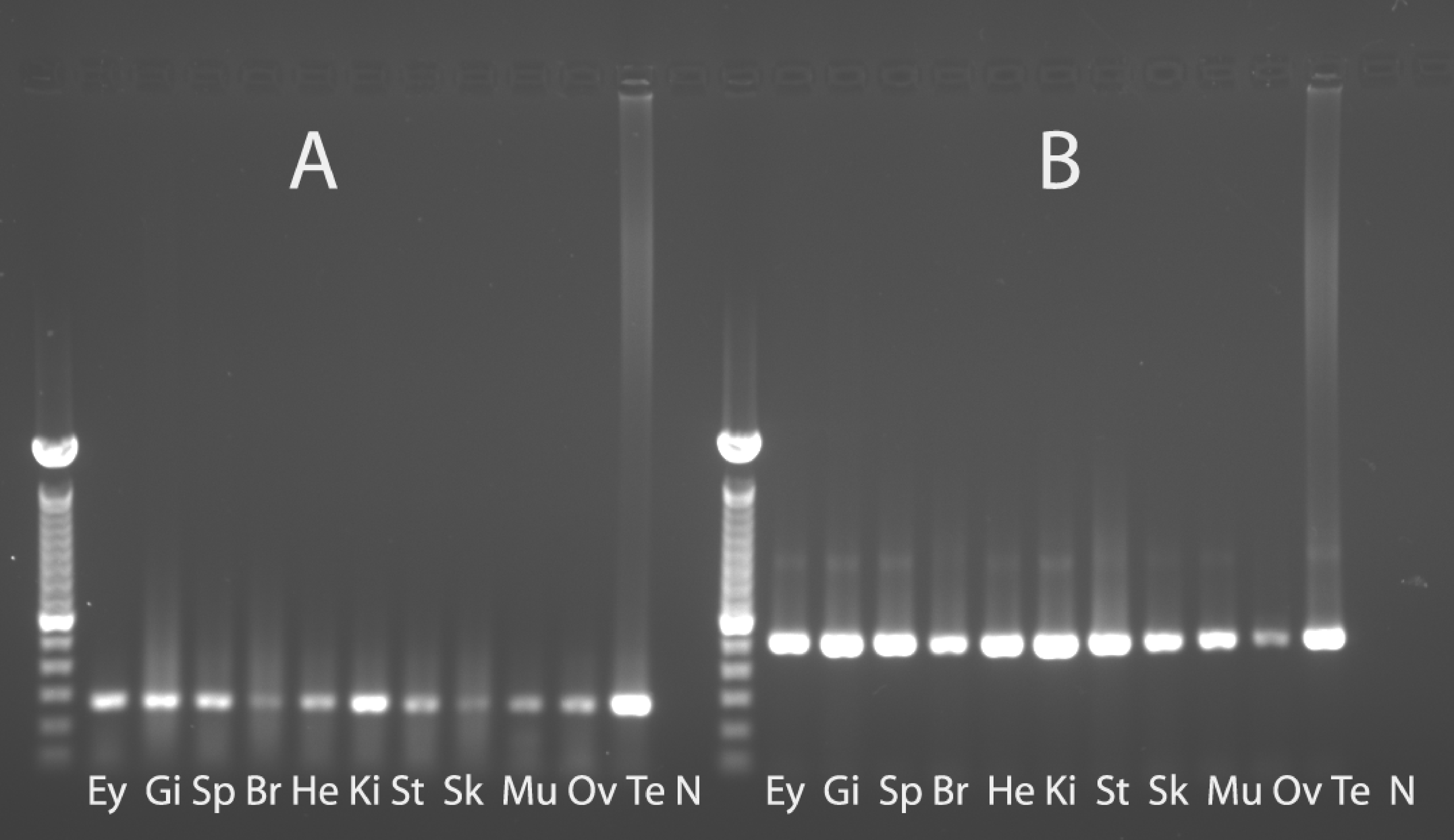
A
B
Ey Gi Sp Br He Ki St Sk Mu Ov Te N
Ey Gi Sp Br He Ki St Sk Mu Ov Te N
