# Supplementary figures and images for: Characterization and localization of cyclin B3 transcript in both oocyte and spermatocyte of the rainbow trout (Oncorhynchus mykiss)
Source: PeerJ. 2019 Jul 24;7:e7396. doi: 10.7717/peerj.7396 (PMC6660826; doi:10.7717/peerj.7396)

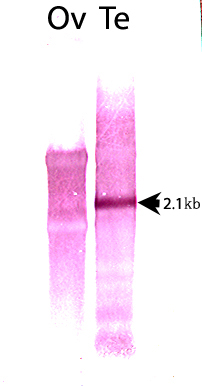

Supplement: Supplemental Information 3 — The detection of an unique form of CB3 mRNA, of approximately 2.1 kb (arrow). [file peerj-07-7396-s003.jpg]

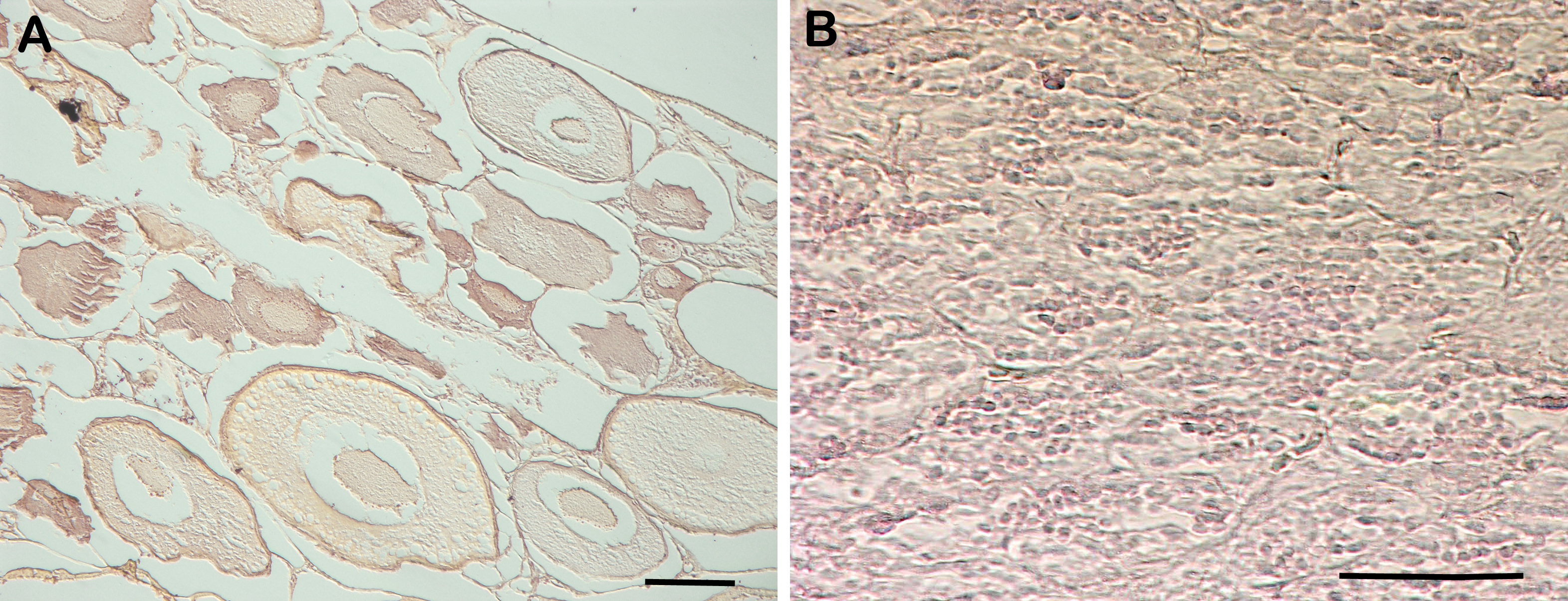

Supplement: Supplemental Information 4 [file peerj-07-7396-s004.jpg]
